# Gamma-Oryzanol Attenuates Aortic Valve Interstitial Cell Calcification via Suppression of BMP2-SMAD and MAPK Signaling Pathways

**DOI:** 10.3390/biom16010107

**Published:** 2026-01-08

**Authors:** Mausam Thapa, Saugat Shiwakoti, Dalseong Gong, Ju-Young Ko, Yeon-Hyang Gwak, Min-Ho Oak

**Affiliations:** 1 College of Pharmacy, Mokpo National University, Muan 58554, Republic of Korea; mausamthapa@mokpo.ac.kr (M.T.);; 2 Convergence Center for Green Anti-Aging Research, Mokpo National University, Muan 58554, Republic of Korea

**Keywords:** CAVS, γ-ORZ, antioxidant, BMP2-SMAD1/5/9 pathway, MAPKs

## Abstract

Calcific aortic valve stenosis (CAVS) is a progressive cardiovascular disease associated with oxidative stress-driven osteogenic differentiation of valvular interstitial cells (VICs), yet no pharmacological therapy can prevent its progression. γ-oryzanol (γ-ORZ), a rice bran-derived phytosteryl ferulate, exhibits potent antioxidative and anti-inflammatory activities that may counteract valvular calcification. Here, we show that γ-ORZ markedly attenuates PCM-induced intracellular ROS elevation, osteogenic differentiation, and calcium phosphate deposition in porcine VICs (pVICs). In addition, RT-qPCR and Western blot analyses revealed significant downregulation of calcification markers (RUNX2, OPN, BMP2), along with suppressed SMAD1/5/9 transcription and phosphorylation, decreased p38/ERK MAPK activation, and reduced ALP activity. Collectively, these findings indicate that γ-ORZ mitigates oxidative stress-mediated valvular calcification by inhibiting both canonical and non-canonical BMP2-SMAD/MAPK signaling, suggesting its potential as a medicinal candidate for early intervention in CAVS.

## 1. Introduction

Calcific aortic valve stenosis (CAVS) is a progressive valvular heart disease that predominantly affects aging populations worldwide. Its prevalence rises to approximately 25% in individuals over 65 years and nearly 50% in those over 85 [1]. In addition to aging, cellular senescence and multiple etiological factors contribute to CVAS, encompassing acquired conditions such as hypertension, diabetes, chronic kidney disease, and smoking, as well as congenital abnormalities, particularly bicuspid aortic valve (BAV) disease [2,3,4]. The disease begins with an asymptomatic phase of aortic valve sclerosis, progressing to pathological calcification of the valve leaflets, which increases stiffness and obstructs left ventricular outflow [5]. Advanced CAVS contributes to heart failure, reduced quality of life, and greater demand for medical intervention [6]. Currently, surgical or transcatheter valve replacement is the only definitive treatment, but these approaches carry procedural risks, high costs, and limitations in certain patient populations [7,8]. This highlights the need for alternative therapeutic strategies that target disease progression.

The complex progression of CAVS can be simplified as a sequence from endothelial damage and inflammation, to fibrosis and osteogenic differentiation of valvular interstitial cells (VICs), and finally to valve calcification [9]. It involves an intricate interplay of mechanical, cellular, and molecular maladaptation in the valve leaflets, influenced by factors such as mechanical stress, aging, and high phosphate levels, often driven by increased ROS production [10]. Elevated ROS, including superoxide, hydrogen peroxide, and hydroxyl radicals, are reported to induce oxidative stress in vascular and valvular tissues [11]. There are currently no effective medical therapies for CAVS. Although statins lower LDL levels, they do not slow calcific progression [12]. Consequently, alternative LDL-lowering agents such as PCSK9 and cholesteryl ester transfer protein (CETP) inhibitors, as well as repurposed drugs like Angiotensin II Receptor Blockers (ARBs) and DPP-4 inhibitors, are under investigation; however, all require further clinical trials to determine their therapeutic benefits [13,14,15]. Therefore, a deeper understanding of the underlying mechanisms and identification of therapeutic agents that slow VIC osteogenic differentiation are essential.

Natural compounds and nutraceuticals with antioxidant and anti-inflammatory properties are increasingly recognized as potential preventive candidates for CAVS [16]. Several polyphenolic compounds, including gallic acid, resveratrol, curcumin, and quercetin, have been reported to mitigate calcification in cellular models [17,18,19]. One of the dietary byproducts rich in bioactive molecules, rice bran extract (RBE) confers antioxidant and anti-inflammatory benefits while improving lipid metabolism and supporting liver and cardiovascular health [20]. Structurally similar to the polyphenols, γ-oryzanol (γ-ORZ), a major component of RBE, is a natural blend of ferulic acid esters of phytosterols and triterpene alcohols, primarily derived from rice bran oil, and is widely recognized for its antioxidant and anti-inflammatory properties [21]. Also, it has been reported to have lipid-lowering, hepatoprotective, neuroprotective, and metabolic regulatory effects, supporting cardiovascular performance and cellular integrity [22,23,24]. Moreover, γ-ORZ has been shown to mitigate oxidative stress driven endothelial senescence and dysfunction while restoring normal proliferative capacity in porcine coronary artery endothelial cells exposed to fine dust [25]. Therefore, understanding the mechanistic effects of γ-ORZ on calcific pathways may reveal its utility as a dietary or adjunctive therapeutic approach for CAVS. This study aimed to evaluate the anti-calcific potential of γ-ORZ in Pro calcifying media (PCM) induced porcine valve interstitial cells (pVICs) and to elucidate the molecular mechanisms underlying its effects.

## 2. Materials and Methods

### 2.1. VICs Isolation, Culture, and Treatment

Porcine hearts were obtained postmortem from a local commercial slaughterhouse in Mokpo, Republic of Korea, which operates by the U.S. Department of Agriculture’s Animal Cruelty and Slaughter Act guidelines. As such, the study was exempt from institutional animal ethics committee approval. Within 30 min of sacrifice, the hearts were transported to the laboratory in ice-cold Krebs bicarbonate solution (in mmol/L: NaCl 119, KCl 4.7, KH_2_PO_4_ 1.18, MgSO_4_ 1.18, CaCl_2_ 1.25, D-glucose 11, and NaHCO_3_ 1.25; pH 7.4) and maintained at 4 °C.

Upon arrival, aortic valve leaflets were dissected under sterile conditions and washed with cold 1× phosphate-buffered saline (PBS). The tissues were then subjected to an initial digestion in type I collagenase solution (600 µg/mL, Worthington, Lakewood, NJ, USA) for 15 min at 37 °C in 5% CO_2_. To remove the endothelial layer, the leaflet surfaces were gently swabbed with a sterile cotton applicator. This was followed by overnight incubation in the same collagenase solution at 37 °C, 5% CO_2_ to ensure complete digestion. The resulting cell suspension was filtered using cell strainer (Falcon^®^ 100 µm; Corning, NY, USA), and cultured in T75 flasks using Dulbecco’s Modified Eagle Medium (DMEM; Gen Depot, Katy, TX, USA) supplemented with 5% fetal bovine serum (FBS), 100 U/mL penicillin, 100 U/mL streptomycin, and 250 μg/mL fungizone. Cultures were maintained until reaching the desired confluence.

To induce calcification, confluent pVICs were exposed to a pro-calcifying medium (PCM) consisting of standard culture medium supplemented with 2 mM sodium dihydrogen phosphate (pH 7.4) and 50 µg/mL ascorbic acid. Cells were treated with γ-Oryzanol (γ-ORZ; was purchased from Tokyo Chemical Industry, Tokyo, Japan; CAS RN-11042-64-1) in PCM for 7 days, with media refreshed every two days. The experimental conditions were divided as follows: a control condition using standard control medium (CM), a PCM condition treated with pro-calcifying medium alone, and PCM conditions supplemented with γ-ORZ at concentrations of 0.1, 1, and 10 μM, respectively.

### 2.2. Cell Viability Assay

pVICs were seeded in 96-well plates at a density of 1 × 10^4^ cells per well and treated with different concentrations of γ-ORZ (0.1, 1, 3, and 10 µM) for 24 and 72 h. Cell viability was assessed using the CellTiter 96^®^ Aqueous One Solution Cell Proliferation Assay (Promega Corporation, Madison, WI, USA). Following treatment, 20 µL of the MTS tetrazolium reagent was added to each well, and the cells were incubated for 2–3 h at 37 °C in a 5% CO_2_ atmosphere. Absorbance was measured at 490 nm using an Enspire Multilabel Reader (PerkinElmer, Inc., Waltham, MA, USA) to quantify cell viability.

### 2.3. Calcification Evaluation

Calcification levels were assessed using Alizarin Red S staining. 40 mM solution of Alizarin Red S (Sigma-Aldrich, St. Louis, MO, USA) was prepared in triple-distilled water, and the pH was adjusted to 4.1–4.2 using 10% ammonium hydroxide (Bio solution, Seoul, Republic of Korea). pVICs were seeded in 96-well plates and cultured for 10 days in PCM, either alone or supplemented with various concentrations of γ-ORZ (0.1, 1, and 10 μM). The control group was maintained in CM without any treatment. Following incubation, cells were fixed with 4% paraformaldehyde (Thermo Scientific, Franklin, MA, USA) and stained with the Alizarin Red S solution for 20 min at room temperature. After two thorough washes, images were captured to visually evaluate calcification. For quantification, cells were treated with 10% cetylpyridinium chloride (Sigma-Aldrich) for 1 h to elute the bound dye. The absorbance of the extracted Alizarin Red S was measured at 550 nm using an Enspire Multilabel Reader (Perkin Elmer Ltd.).

### 2.4. Western Blot Analysis

pVICs were cultured and treated for 7 days under various experimental conditions, then collected and lysed for protein extraction. Equal amounts of protein (25 µg per lane) were loaded onto SDS-polyacrylamide gels for electrophoresis and subsequently transferred onto polyvinylidene fluoride (PVDF) membranes. The membranes were then blocked with 5% Bovine serum albumin (BSA; Prod. code BSAS-AU, Bovostar™ Premium BSA, Bovogen Biologicals, Keilor East, Australia) and incubated overnight at 4 °C with the respective primary antibodies (Table S1). This was followed by an hour incubation at room temperature with horseradish peroxidase (HRP) conjugated anti-mouse or anti-rabbit IgG secondary antibodies (1:10,000; Cell Signaling Technology, Danvers, MA, USA). Protein signals were detected using the Super Signal™ West Pico PLUS enhanced chemiluminescence substrate (Thermo Scientific). Expression levels of target proteins were evaluated based on at least three independent experiments. Protein band intensities were measured using ImageJ (Version 1.53h; National Institutes of Health, Bethesda, MD, USA), and the expression levels were standardized to β-actin.

### 2.5. Evaluation of ROS Levels

To evaluate intracellular reactive oxygen species (ROS), cells were treated with the redox-sensitive, cell-permeable dye DCF-DA, followed by qualitative assessment using confocal fluorescence microscopy and quantitative measurement using flow cytometry. For confocal fluorescence imaging, pVICs were seeded in Nunc™ Lab-Tek™ II 2-well Chamber Slides (Thermo Fisher Scientific, USA) at a density of 5 × 10^4^ cells per well and maintained for 7 days in PCM supplemented with γ-ORZ (0.1, 10 µM). Cells were washed with 1× PBS and incubated with DCF-DA (10 μM) for 20 min at 37 °C, 5% CO_2_, rinsed with 1× PBS, and subsequently counterstained with DAPI (10 μM) for 10 min. The cells were then fixed with 4% formaldehyde for 10 min, rinsed once more, and the slides were then prepared for analysis. Fluorescence signals were detected with an FITC filter and the images were captured using a ZEISS LSM980 confocal microscope (ZEISS, Oberkochen, Germany). For flow cytometry analysis, pVICs were seeded in 6-well plates at a density of 1 × 10^5^ cells per well and treated for 7 days with varying concentrations of γ-ORZ (0.1, 1, and 10 µM) in PCM. Cells were washed with 1× PBS and incubated with DCF-DA (10 μM) for 20 min at 37 °C, 5% CO_2_, rinsed with 1× PBS then fixed with 4% formaldehyde solution for 10 min. The cells were rinsed again, trypsinized, and collected into an Eppendorf tube containing culture medium. They were then centrifuged at 4 °C, washed twice, and finally resuspended in 1× PBS. Fluorescence intensity was quantified using a Beckman Coulter Cytoflex flow cytometer (Beckman Coulter, Bera, CA, USA), and the data were processed with CytExpert 2.4. Fluorescence fold changes were calculated relative to the mean intensity of the PCM.

### 2.6. RT-qPCR Assay

Total RNA was extracted using TRIzol reagent (Takara Bio, Shiga, Japan), and cDNA was synthesized following the manufacturer’s protocol with the PrimeScript 1st Strand cDNA Synthesis Kit (Takara Bio). Gene expression was quantified by RT-qPCR using TB Green Premix Ex Taq II (Takara Bio) on a Bio-Rad CFX384 Touch Real-Time PCR system (Bio-Rad, Hercules, CA, USA), and relative expression levels were determined using the 2−ΔΔCt method. Primers were designed based on NCBI reference sequences and assessed for specificity and amplification efficiency; the corresponding sequences are detailed in Supplementary Table S2.

### 2.7. Statistical Analysis

Results are presented as the mean ± standard error of the mean (SEM). Statistical analyses were conducted using either one-way ANOVA followed by Tukey’s post hoc test or two-way ANOVA with Bonferroni post hoc correction. All analyses were performed with Prism software (v8.0, GraphPad, San Diego, CA, USA), and *p*-values less than 0.05 were considered statistically significant.

## 3. Results

### 3.1. γ-ORZ Reduces PCM-Induced Calcification of VICs

**Figure 1:**
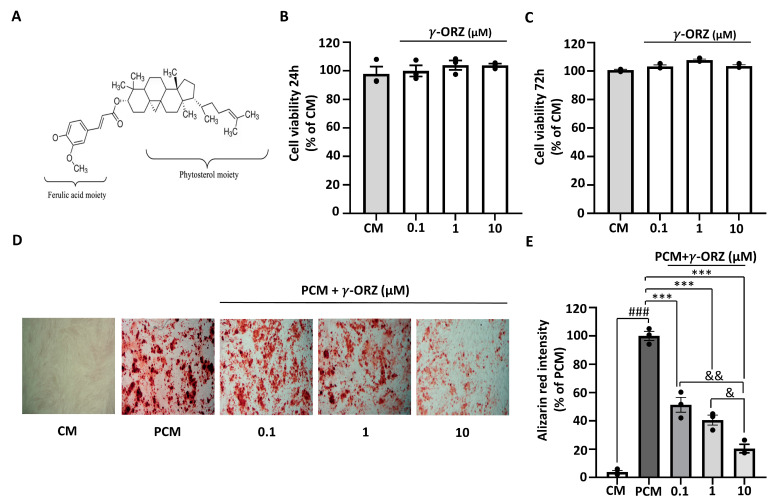
Effect of γ-oryzanol (γ-ORZ) on phosphate-induced calcification in VICs. (**A**) Molecular structure of γ-ORZ. (**B**,**C**) Cell viability of VICs treated with different concentrations of γ-ORZ (0.1, 1, 10 μM) for 24 h (B) and 72 h (**C**), determined by the MTS (3-(4,5-dimethylthiazol-2-yl)-5-(3-carboxymethoxyphenyl)-2-(4-sulfophenyl)-2H-tetrazolium) assay. Results are expressed as the mean ± standard error of the mean (SEM) (*n* = 3). (**D**) Representative images of Alizarin Red S staining showing calcium deposition in VICs cultured under control media (CM), pro-calcifying media (PCM), or PCM supplemented with γ-ORZ (0.1, 1, 10 μM) for 7 days. Red staining indicates calcium deposition (Original magnification: 100×). (**E**) Relative quantification of Alizarin Red S staining intensity by cetylpyridinium chloride assay. Results are expressed as the mean ± SEM (*n* = 3). ### *p* < 0.001 vs. CM; *** *p* < 0.001 vs. PCM; & *p* < 0.05 and && *p* < 0.01 between γ-ORZ treatment groups (0.1, 1, and 10 μM).

The molecular structure of γ-ORZ is illustrated in Figure 1A. Cell viability was assessed in VICs using the MTT assay at 24 and 72 h to determine cytotoxicity and appropriate treatment doses. γ-ORZ was treated at different concentrations (0.1, 1, and 10 μM). Results indicated that γ-ORZ did not affect cell viability at concentrations up to 10 μM (Figure 1B,C). PCM containing sodium dihydrogen phosphate and ascorbic acid is widely used as an in vitro model to induce VIC activation and calcification [26]. To evaluate the role of γ-ORZ in PCM-induced calcification, VICs were cultured in PCM with or without γ-ORZ (0.1, 1, and 10 μM) for 7 days. Thereafter, VICs were stained with Alizarin Red S staining followed by cetylpyridinium assay for quantification. Figure 1D images represents Alizarin Red-stained calcium phosphate deposition in VICs cultured in PCM compared with CM, with PCM-treated cells exhibiting significantly higher staining. Furthermore, γ-ORZ treatment resulted in an overall significant, dose-dependent reduction in Alizarin Red staining intensity in VICs cultured in PCM (Figure 1D,E). Moreover, statistically significant reductions were observed between the higher and lower γ-ORZ doses (0.1 vs. 10 μM and 1 vs. 10 μM) (Figure 1E). These results demonstrate that γ-ORZ reduce the rate of PCM-induced calcification in VICs.

### 3.2. γ-ORZ Suppresses Calcification Markers Triggered by PCM

**Figure 2:**
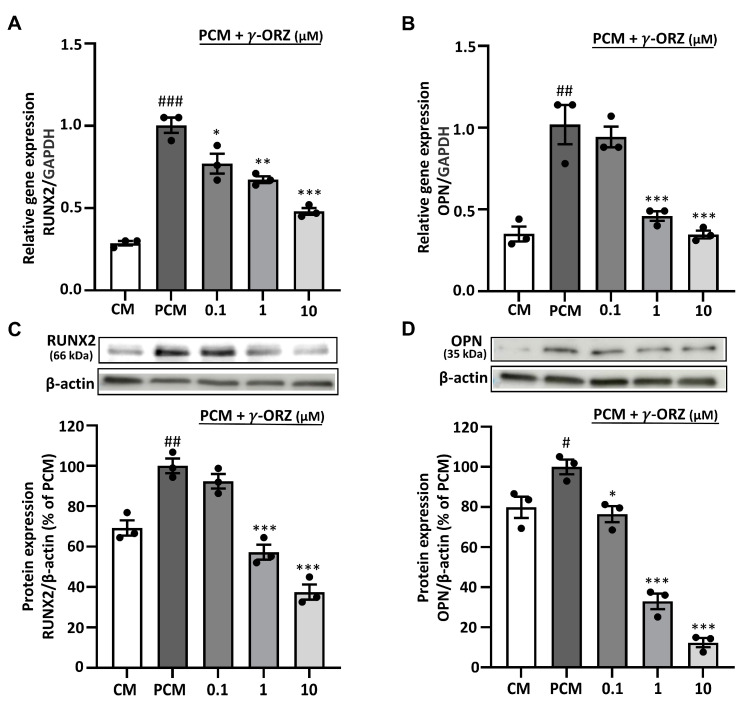
Effect of γ-ORZ on calcification-related gene and protein expression in VICs. (**A**,**B**) Relative mRNA expression levels of *RUNX2* (**A**) and *OPN* (**B**) in VICs cultured under CM, PCM, or PCM supplemented with γ-ORZ (0.1, 1, 10 µM) for 7 days, determined by quantitative real-time PCR and normalized to *GAPDH*. (**C**,**D**) Representative Western blot images and corresponding densitometric analyses showing protein expression of RUNX2 (**C**) and OPN (**D**), normalized to β-actin and expressed as a percentage of the PCM group. Results are expressed as the mean ± standard error of the mean (SEM) (*n* = 3). # *p* < 0.05, ## *p* < 0.01, ### *p* < 0.001 vs. CM; * *p* < 0.05, ** *p* < 0.01, *** *p* < 0.001 vs. PCM. Original images can be found at Supplementary Figure S1.

It is known that PCM promotes calcification, accompanied by elevated expression of osteogenic markers such as runt-related transcription factor 2 (RUNX2) and osteopontin (OPN) [27]. To assess the change in molecular expression of these calcification markers, VICs were cultured for 7 days in PCM in the presence or absence of γ-ORZ (0.1, 1, and 10 μM). The results indicated that PCM-treated cells had significantly higher mRNA and protein levels of RUNX2 and OPN compared to CM (Figure 2). In addition, a significant reduction in the level of calcification markers was observed at both the transcriptional and translational levels in the γ-ORZ-treated groups at higher doses (1 and 10 μM), whereas the effects were inconsistent at the lower dose (0.1 μM) (Figure 2). In combination with the Alizarin Red study, the results indicate that γ-ORZ inhibits PCM-induced calcification by suppressing calcification marker expression at both the gene and protein expression levels.

### 3.3. γ-ORZ Decreases PCM-Induced Oxidative Stress and Upregulation of ALP

**Figure 3:**
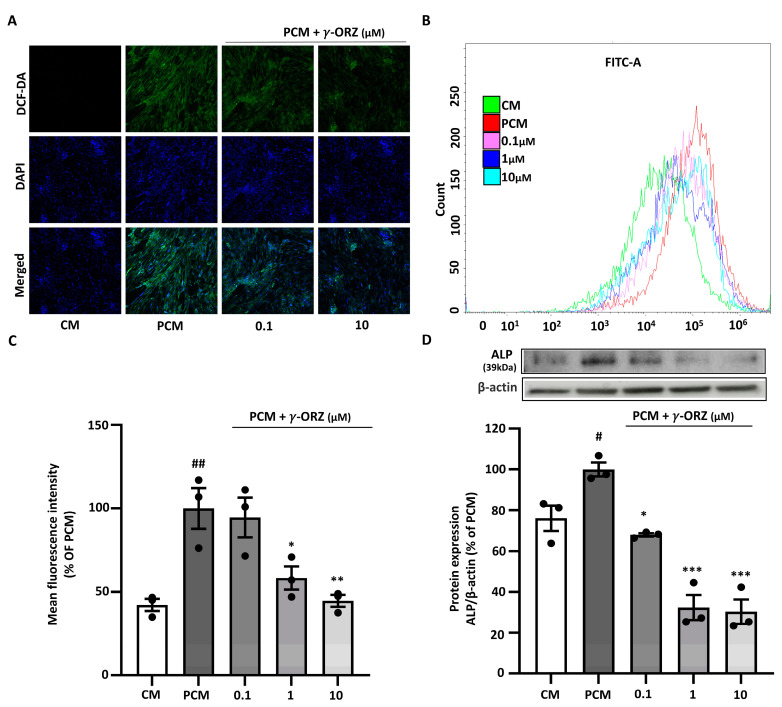
Effect of γ-ORZ on oxidative stress and alkaline phosphatase (ALP) expression in VICs cultured with pro-calcifying conditions. (**A**) Representative fluorescence images of intracellular ROS levels in VICs cultured under CM or PCM with or without γ-ORZ (0.1 and 10 µM) for 7 days. Cells were stained with DCF-DA (green) for ROS and DAPI (blue) for nuclei (Original magnification: 200×). (**B**) Flow cytometry histograms showing DCF-DA fluorescence intensity in each treatment group (γ-ORZ: 0.1, 1, and 10 µM). (**C**) Quantitative analysis of mean fluorescence intensity expressed as a percentage of the PCM group. (**D**) Representative Western blot image and corresponding densitometric analyses showing protein expression of ALP, normalized to β-actin and expressed as a percentage of the PCM group. Results are presented as the mean ± standard error of the mean (SEM) (*n* = 3). # *p* < 0.05, ## *p* < 0.01 vs. CM; * *p* < 0.05, ** *p* < 0.01, *** *p* < 0.001 vs. PCM. Original images can be found at Supplementary Figure S1.

PCM is known to elevate intracellular ROS, which facilitates calcification by promoting oxidative stress [28]. Moreover, elevated ROS levels are often associated with increased alkaline phosphatase (ALP) expression, thereby promoting vascular calcification [29]. ROS levels were assessed using the fluorescent probe DCF-DA for qualitative (confocal microscopy) and quantitative (flow cytometry) analysis to evaluate the effect of γ-ORZ on PCM-induced ROS production. VICs treated with PCM exhibit increased green fluorescence compared to control cells, indicating elevated intracellular ROS; nuclei were counterstained with DAPI (blue) Figure 3A. Subsequently, treatment with γ-ORZ at both the lower and higher doses (0.1 and 1 μM) resulted in a prominent reduction in DCF-DA fluorescence intensity (3A). Correspondingly, flow cytometry assessment indicated a significant reduction in ROS levels, consistent with the microscopic image analysis (Figure 3B,C). Additionally, Western blot analysis (Figure 3D) showed a significant dose-dependent reduction in ALP protein expression in VICs with γ-ORZ treatment. Taken together, these results suggest that γ-ORZ inhibits calcification in VICs, at least in part, by mitigating oxidative stress and reducing oxidative stress mediated downstream proteins such as ALP.

### 3.4. γ-ORZ Suppresses Canonical BMP2/SMAD Signaling

**Figure 4:**
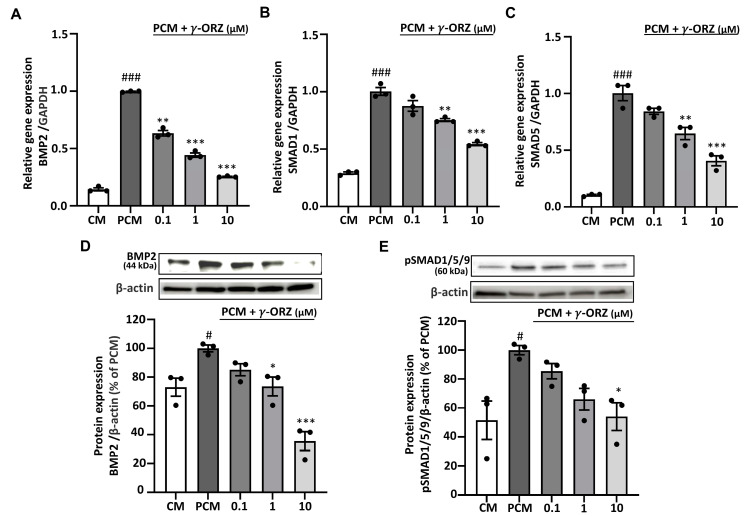
Effect of γ-ORZ on canonical BMP2/SMAD signaling in VICs. (**A**–**C**) Relative mRNA expression levels of *BMP2* (**A**), *SMAD1* (**B**), and *SMAD5* (**C**) in VICs cultured under CM, PCM, or PCM supplemented with γ-ORZ (0.1, 1, or 10 µM) for 7 days, as determined by quantitative real-time PCR and normalized to *GAPDH*. (**D**,**E**) Representative Western blot images and corresponding densitometric analyses showing BMP2 (**D**) and phosphorylated SMAD1/5/9 (p-SMAD1/5/9) (**E**) protein expression. Protein levels were normalized to β-actin and expressed as a percentage of the PCM group. Results are expressed as the mean ± standard error of the mean (SEM) (*n* = 3). # *p* < 0.05, ### *p* < 0.001 vs. CM; * *p* < 0.05, ** *p* < 0.01, *** *p* < 0.001 vs. PCM. Original images can be found at Supplementary Figure S2.

BMP-2 is mainly known for its involvement in ossification-like processes, which resembles the osteoblastic differentiation of VICs during calcification and acts as a crucial mediator in AVS progression [30]. In addition, ROS play a critical role in BMP-2 signaling necessary for early osteoblast differentiation [31]. Moreover, canonical BMP2 signaling is mediated by the phosphorylation of SMAD proteins such as SMAD1, SMAD5, and SMAD9, early markers of the pro-calcific signaling cascade [32]. Thus, to evaluate the role of γ-ORZ in PCM-induced BMP2 signaling during VIC calcification, *SMAD1*, *SMAD5* and *BMP2* gene expression were assessed. Furthermore, BMP2 and pSMAD1/5/9 protein levels were measured after 7 days of treatment with PCM, with or without γ-ORZ (0.1, 1, and 10 µM). The results showed that in PCM-treated VICs, gene expression of *BMP2*, *SMAD1* and *SMAD5* (Figure 4A–C) and protein levels of BMP2 and pSMAD1/5/9 (Figure 4D,E) were significantly increased compared to the control media. Concurrently, γ-ORZ treatment led to a dose dependent reduction in *BMP-2*, *SMAD1* and *SMAD5* mRNA levels, as well as in the protein expression of BMP-2 and pSMAD1/5/9 (Figure 4). Cumulatively, these findings suggest that γ-ORZ inhibits the canonical BMP-2/SMAD signaling pathway and may attenuate the early progression of calcification in VICs.

### 3.5. γ-ORZ Suppresses Non Canonical BMP2/SMAD-MAPK Signaling

**Figure 5:**
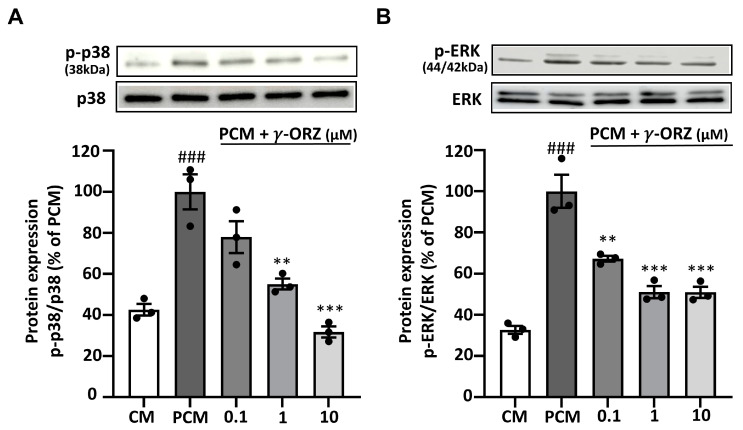
Effects of γ-ORZ on p38 and ERK MAPK signaling in VICs. (**A**,**B**) Representative Western blot images and corresponding densitometric analyses showing phosphorylated p38 (p-p38) and total p38 (**A**), and phosphorylated ERK (p-ERK) and total ERK (**B**) in VICs cultured under CM, PCM, or PCM supplemented with γ-ORZ (0.1, 1, or 10 µM) for 7 days. Phosphorylated protein levels were normalized to their respective total protein levels (p-p38/p38 and p-ERK/ERK) and expressed as a percentage of the PCM group. Results are expressed as the mean ± standard error of the mean (SEM) (*n* = 3). ### *p* < 0.001 vs. CM; ** *p* < 0.01, *** *p* < 0.001 vs. PCM. Original images can be found at Supplementary Figure S3.

BMP2 signaling has also been reported to activate non-canonical pathways, particularly p38 and ERK MAPKs [33]. Moreover, these MAPKs are reported to amplify calcification by upregulating osteogenic signaling and Runx2-mediated transcription [34]. On this basis, the phosphorylation of p38 and ERK was tested to determine the effect of γ-ORZ on PCM-induced non-canonical MAPK signaling in VICs. Western blot analysis illustrated that PCM treatment significantly increased p-p38 and p-ERK levels in VICs compared with control media (Figure 5A,B). Meanwhile, treatment with γ-ORZ significantly reduced PCM-stimulated activation of p38 and ERK. Although a significant overall reduction in p-ERK levels was observed (Figure 5B), no further reduction occurred between the 1 and 10 µM γ-ORZ treated groups. These findings highlight that γ-ORZ block MAPKs activation, potentially limiting BMP2-mediated non-canonical pro-calcific signaling.

## 4. Discussion

These results demonstrate that γ-ORZ reduces PCM-induced calcification, at least in part, by mitigating oxidative stress mediated calcification markers, including RUNX2 and OPN, at both transcriptional and translational levels. Additionally, γ-ORZ inhibits both canonical and noncanonical BMP2/SMAD signaling pathways. Overall, our findings suggest that γ-ORZ attenuates PCM-induced calcification in VICs by suppressing ROS-mediated BMP2/SMAD and p38/ERK MAPK pathways and may serve as a therapeutic adjunct to prevent CAVS.

CAVS is a progressive and multifactorial disorder, largely driven by elevated ROS and chronic inflammation [35]. There are both congenital and non-congenital factors that contribute to the progression of CAVS, including senescence, hypertension, diabetes, chronic kidney disease, and smoking, as well as structural cardiac anomalies, particularly BAV disease [4,36]. In CAVS, VICs throughout the valvular layers become activated and undergo osteogenic differentiation, as indicated by increased expression of markers such as runt-related transcription factor 2 (RUNX2) and osteopontin (OPN) [37]. This phenotypic transition promotes inflammatory cell recruitment and cytokine release, including IL-6 and TNF-α, and activate pathways such as Wnt/β-catenin, NF-κB, and RANK/RANKL/OPG [38,39]. These signaling pathways upregulate the expression of osteogenic genes, including *RUNX2* and *OPN*, driving VICs toward osteogenic differentiation and ultimately leading to valve stiffening through calcification and matrix mineralization [40]. These structural changes reduce leaflet compliance, impair systolic ejection, and can lead to increased ventricular pressure, hypertrophy, heart failure, and reduced cardiac output [41]. Despite ongoing research, there are no approved drugs capable of preventing or reversing CAVS progression, leaving surgery as the primary effective treatment [8]. Alternative LDL-lowering agents, including PCSK9 and CETP inhibitors, as well as repurposed drugs like ARBs and DPP-4 inhibitors, are under investigation, but their efficacy requires further clinical validation [13,14]. Hence, further research into the underlying mechanisms and the development of effective therapies is urgently needed for the prevention of this disease.

Among various beneficial nutraceuticals, γ-ORZ has a long history of medicinal and commercial use, ranging from applications in cosmetics to recognized benefits for vascular health [42]. γ-ORZ ‘s beneficial properties are attributed to its polyphenolic structure, which consists of ferulic acid esters derived predominantly from rice bran oil [43]. It is well recognized for its antioxidant and anti-inflammatory properties, with numerous studies demonstrating its ability to attenuate oxidative stress [22]. Its robust ROS-scavenging activity is linked to enhanced antioxidant activity (GR, GSH-Px, CAT, SOD, PON1) and suppression of inflammatory mediators (NF-κB, IL-1β, TNF-α) [42]. γ-ORZ has been shown to have potential in mitigating diseases such as Parkinson’s disease, diabetes, and cancer, involving pathways such as Akt, nitric oxide (NO), NF-κB, PPARγ, MAPKs, and Wnt/β-catenin, all of which are directly linked to its antioxidant and anti-inflammatory activities [42,44]. Furthermore, γ-ORZ has been shown to exert vascular protective effects by alleviating fine dust-induced endothelial senescence and dysfunction, as well as by suppressing endothelial adhesion molecule expression and leukocyte adhesion [25,45]. Therefore, these factors underscore the need to explore the mechanistic pathways through which γ-ORZ may exert preventive effects in CAVS.

In this study, we examined for the first time the anti-calcific potential of γ-ORZ in PCM induced VICs. Our findings indicate that γ-ORZ attenuates valvular calcification, partly through suppression of the BMP2/SMAD axis and the associated MAPK signaling pathways.

Calcification promoted by PCM is associated with elevated intracellular ROS and upregulation of osteogenic markers such as RUNX2 and OPN, resulting in oxidative stress [46]. PCM induced calcification serves as a reliable in vitro model for investigating tissue mineralization, where calcium deposition can be assessed using Alizarin Red staining [47]. In our study, we observed that treatment with γ-ORZ reduced the intensity of Alizarin Red stained calcium nodules and downregulated both transcriptional and translational expression of key calcification markers RUNX2 and OPN. Additionally, our study showed that γ-ORZ markedly reduced DCF-DA fluorescence in PCM-treated VICs, indicating decreased ROS level consistent with its reported antioxidant activity. Also, Elevated ROS levels are frequently associated with increased ALP activity in vascular calcification, mediated via pathways such as NF-κB/p52 in human aortic endothelial cells [48]. ALP contributes to the progression of calcification by hydrolyzing pyrophosphate, a potent inhibitor of mineral deposition [49]. We found that γ-ORZ treatment reduced oxidative stress and consequently suppressed ALP expression under pro-calcifying conditions. These effects are likely mediated through γ-ORZ’s dual capacity to scavenge ROS and modulate key transcriptional pathways, thereby disrupting both oxidative and osteogenic signaling [50]. Moreover, the sterol-derived moieties present in γ-ORZ may further chelate divalent metal ions (e.g., Zn^2+^, Mg^2+^) essential for ALP catalytic activity, providing an additional layer of inhibition at the enzymatic level [51,52]. Hence, these findings suggest that γ-ORZ has the potential to attenuate oxidative stress mediated VICs calcification and may serve as a preventive strategy against CAVS.

Amid the wide-ranging effects of elevated ROS, there is increased expression of RUNX2, OPN, and BMP2, which are key mediators of valvular calcification [53]. CAVS resonates the process of bone formation, characterized by activation of BMP2 signaling [30]. BMP2 signaling regulates osteogenic differentiation through both canonical and noncanonical pathways [54]. In the canonical pathway, BMP2 binds to type II BMP receptors (BMPR2), which recruit and activate type I receptors (BMPR1) [54,55]. Activated BMPR1 phosphorylates SMAD1/5/9 at their C-terminal regions, allowing these SMADs to form complexes with SMAD4 and translocate to the nucleus, where they induce transcription of osteogenic genes such as *RUNX2*, *OPN*, *BMP2*, *ALP* [19,55,56]. PCM induces BMP2 signaling, which subsequently acts through both autocrine and paracrine mechanisms to further promote osteogenic differentiation and calcification [57]. In our study, γ-ORZ treatment resulted in a clear reduction in the transcription of *BMP2,* SMAD1 and *SMAD5*, accompanied by decreased protein expression of BMP2 and pSMAD1/5/9, indicating suppression of BMP2 mediated signaling activity. However, the direct expression or modulation of BMP receptors (BMPR1/2) was not examined in this study and should be further investigated to clarify receptor level regulation by γ-ORZ. Nevertheless, our results suggest that γ-ORZ attenuates this canonical BMP2/SMAD-dependent osteogenic drive in VICs, thereby limiting phenotypic transition and calcific matrix formation.

**Figure 6:**
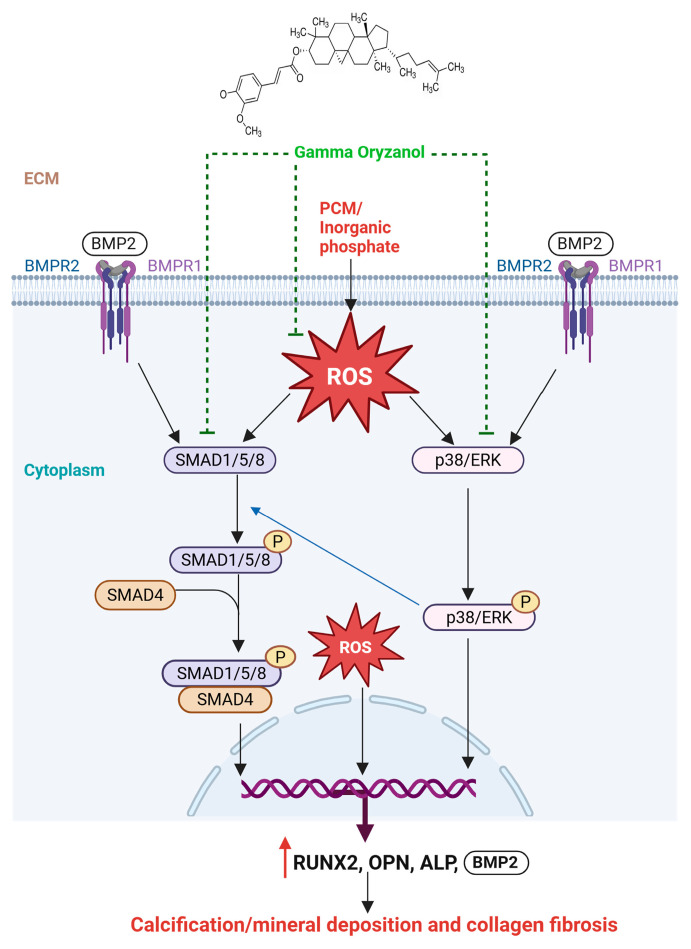
Proposed Mechanism of γ-ORZ-Mediated Anti-Calcific Effects. γ-ORZ inhibits BMP2/SMAD and p38/ERK MAPK signaling while reducing oxidative stress, thereby suppressing osteogenic activation and preventing VIC calcification.

In parallel, BMP2 activates noncanonical signaling cascades most notably p38 and ERK MAPKs reported to contribute to calcification [58]. MAPKs are directly activated via receptor tyrosine kinases (RTKs), which recruit SH2-domain-containing proteins to facilitate tyrosine phosphorylation [59]. In contrast, BMP2R-mediated activation of p38/ERK requires recruitment of the adaptor protein GRB2, which contains SH2 domains and serves as a scaffold for ERK signaling [59]. Activated MAPKs have been reported to phosphorylate SMAD1/5/9 at their linker regions unlike canonical activation [55,60,61]. This linker region phosphorylation amplifies RUNX2 transcriptional activity and the expression of other osteogenic genes, thereby reinforcing osteogenic differentiation [60]. In our study, γ-ORZ significantly reduces p38/ERK MAPK activation and may inhibit SMAD1/5/9 linker region phosphorylation, suggesting interference with BMP2-MAPK crosstalk. In addition to BMP2 mediated osteogenic signaling, TGF-β1 sharing functional similarities with BMP family members plays a pivotal role in promoting VIC activation and calcific remodeling during CAVS progression [62]. These ligands are reported to engage overlapping signaling pathways [63], and examining their convergence could further clarify or even synergize with the molecular mechanisms underlying the anti-calcific effects of γ-ORZ. Overall, these results suggest that γ-ORZ suppresses ROS-mediated activation of both canonical and non-canonical BMP2/SMAD and MAPK pathways, thereby at least partially reducing calcification and potentially preventing CAVS (Figure 6).

The experimental approach in this study relied on an in vitro design using PCM-induced pVICs, which may not fully replicate the hemodynamic and inflammatory complexities of CAVS in vivo. Despite general similarities between species, notable interspecies differences persist. pVICs are reported to express higher levels of myofibroblastic markers such as α-SMA, vimentin, and cadherin-2 than hVICs, indicating species-dependent variation in basal activation state, extracellular matrix remodeling activity, and responsiveness to osteogenic and inflammatory cues [64]. These differences may influence the cellular response to γ-ORZ and limit direct extrapolation of our findings to human pathophysiology. Thus, qRT-PCR analysis of myofibroblastic markers (*ACTA2*, *CNN2*) and ECM components (*FN*, *COL1A1*) can be performed to strengthen the phenotypic validation of the isolated cells by providing additional molecular confirmation of VIC activation. In addition, the bioavailability, pharmacokinetics, and long-term safety of γ-ORZ in cardiac tissues also remain to be clarified. Future studies should therefore validate these mechanistic insights in animal models of CAVS and explore potential synergistic effects of γ-ORZ with established antioxidant or anti-inflammatory agents. Moreover, detailed molecular docking and proteomic analyses could help identify specific γ-ORZ binding targets within the BMP2/MAPK axis, strengthening its translational potential as a dietary derived or adjunctive therapy for CAVS.

## 5. Conclusions

In summary, our results support the potential of γ-ORZ as a dietary adjunct to at least partly prevent CAVS by targeting the underlying calcification mechanisms. Its well-known antioxidant properties were consistent with our findings, as γ-ORZ reduced elevated ROS under pro-calcifying conditions. Importantly, we observed a marked reduction in key calcification markers, including RUNX2, OPN, and ALP, at both the mRNA and protein levels, reinforcing the anti-calcific effects of γ-ORZ. Furthermore, our findings reveal that γ-ORZ may, at least in part, attenuate calcification and inhibit CAVS progression by downregulating BMP2/SMAD and p38/ERK MAPK signaling pathways. Overall, our results suggest that γ-ORZ exerts anti-calcific effects through multiple mechanisms including attenuation of oxidative stress, inhibition of BMP2/SMAD and p38/ERK MAPK pathways and suppression of osteogenic enzyme activity and hence carries the potential to be used as an anti-calcific agent in CAVS to slow disease progression.

## Data Availability

All data generated or analyzed during this study are included in the published article and its Supplementary Information files.

## Supplementary Materials

The following supporting information can be downloaded at: https://www.mdpi.com/article/10.3390/biom16010107/s1, Table S1: Antibodies list used for Western blot experiments; Table S2: Primer’s list used for RT-qPCR experiments corresponding to Figure 2A (*RUNX2*), 2B (*OPN*), 4A (*BMP2*), 4B (*SMAD1*) and 4C (*SMAD5*); Figure S1: Representative full-length uncropped Western blot images corresponding to Figure 2C (RUNX2), Figure 2D (OPN) and Figure 3D (ALP); Figure S2: Representative full-length uncropped Western blot images corresponding to Figure 4D (BMP2), Figure 4E (pSMAD1/5/9); Figure S3: Representative full-length uncropped Western blot images corresponding to Figure 5A (p-p38) and Figure 5B (p-ERK).

## Abbreviations

The following abbreviations are used in this manuscript:

CAVS	Calcific aortic valve stenosis
VICs	Valvular interstitial cells
γ-ORZ	γ-Oryzanol
pVICs	Porcine VICs
ROS	Reactive oxygen species
VECs	Valvular endothelial cells
DPPIV	Dipeptidyl Peptidase-4
AGEs	Advanced glycation end-products
RUNX2	Runt-related transcription factor 2
OPN	Osteopontin
BMP2	Bone morphogenetic protein 2
MAPK	Mitogen-activated protein kinase
SMAD	Sma and Mad-related proteins
ALP	Alkaline phosphatase
ERK	Extracellular signal-regulated kinase
CETP	Cholesteryl ester transfer protein
ARBs	Angiotensin II Receptor Blockers
PCM	Pro calcifying media
CM	Control medium
DCF-DA	2′,7′-dichlorofluorescin diacetate
TGF-β1	Transforming growth factor beta 1
