# Supplementary material for: Gamma-Oryzanol Attenuates Aortic Valve Interstitial Cell Calcification via Suppression of BMP2-SMAD and MAPK Signaling Pathways
Source: Biomolecules. 2026 Jan 8;16(1):107. doi: 10.3390/biom16010107 (PMC12838785; doi:10.3390/biom16010107)
Supplement: Supplementary file 1 [file biomolecules-16-00107-s001.zip › biomolecules-4035637-supplementary.pdf]

SUPPLEMENTARY FILE

Gamma-Oryzanol Attenuates Aortic Valve Interstitial Cell Calcification via Suppression of BMP2-SMAD and MAPK Signaling Pathways

Mausam Thapa [1,2], Saugat Shiwakoti [1,2], Dalseong Gong [1,2], Ju-Young Ko [1,2], Yeon-Hyang Gwak [1] and Min-Ho Oak [1,2,*]

[1] College of Pharmacy, Mokpo National University, Muan 58554, Republic of Korea; mausamthapa@mokpo.ac.kr (M.T.)

[2] Convergence Center for Green Anti-Aging Research, Mokpo National University, Muan 58554, Republic of Korea

* Correspondence: mhoak@mokpo.ac.kr; Tel.: +82-61-450-2681

Table S1: ***Antibodies list used for western blot experiments.***

Antibody	Company	Item Number	Dilution Ratio
RUNX2	Abcam	ab76956	1:1000
OPN	Proteintech	22952-I-AP	1:2000
BMP2	Proteintech	66383-1-Ig	1:1000
pSMAD1/5/9	Cell Signaling	#13820	1:1000
P38	Cell Signaling	#9212	1:2000
p-p38	Cell Signaling	#9211	1:1000
ERK	Cell Signaling	#9102	1:3000
p-ERK	Cell Signaling	#4370s	1:4000
ALP	Santa Cruz Biotechnology	sc-365765	1:1000
β-actin	Santa Cruz Biotechnology	sc-47778	1:50,000

Table S2: ***Primer's list used for RT- qPCR experiments corresponding to Figure 2A (RUNX2), 2B (OPN), 4A (SMAD1), 4B (BMP2).***

Gene	Primer	Sequence (5'-3')
RUNX2	Forward	5'-CCC TGA ACT CTG CAC CAA G-3'
	Reverse	5'-TCT GGC TCA AGT AGG AGG GA-3'
OPN	Forward	5'-GGA GGA AAC GGA CGA CTT CAA ACA-3'
	Reverse	5'-GGC TTC GGA TCT GCG GAA CTT C-3
SMAD1	Forward	5'-GCCAACCCGGTGCTGA-3'
	Reverse	5'-CTTCTTTGCGGTGAGGGTCT-3'
SMAD5	Forward	5'-ATTCGAGGCGGTGTATGAGC-3'
	Reverse	5'-TTCACTGTTTACGGCGGTCC-3'
BMP2	Forward	5'-GCACCAGCTTTTCCTTTCTCC-3'
	Reverse	5'-GCTGACACACAACAACAGCG-3'

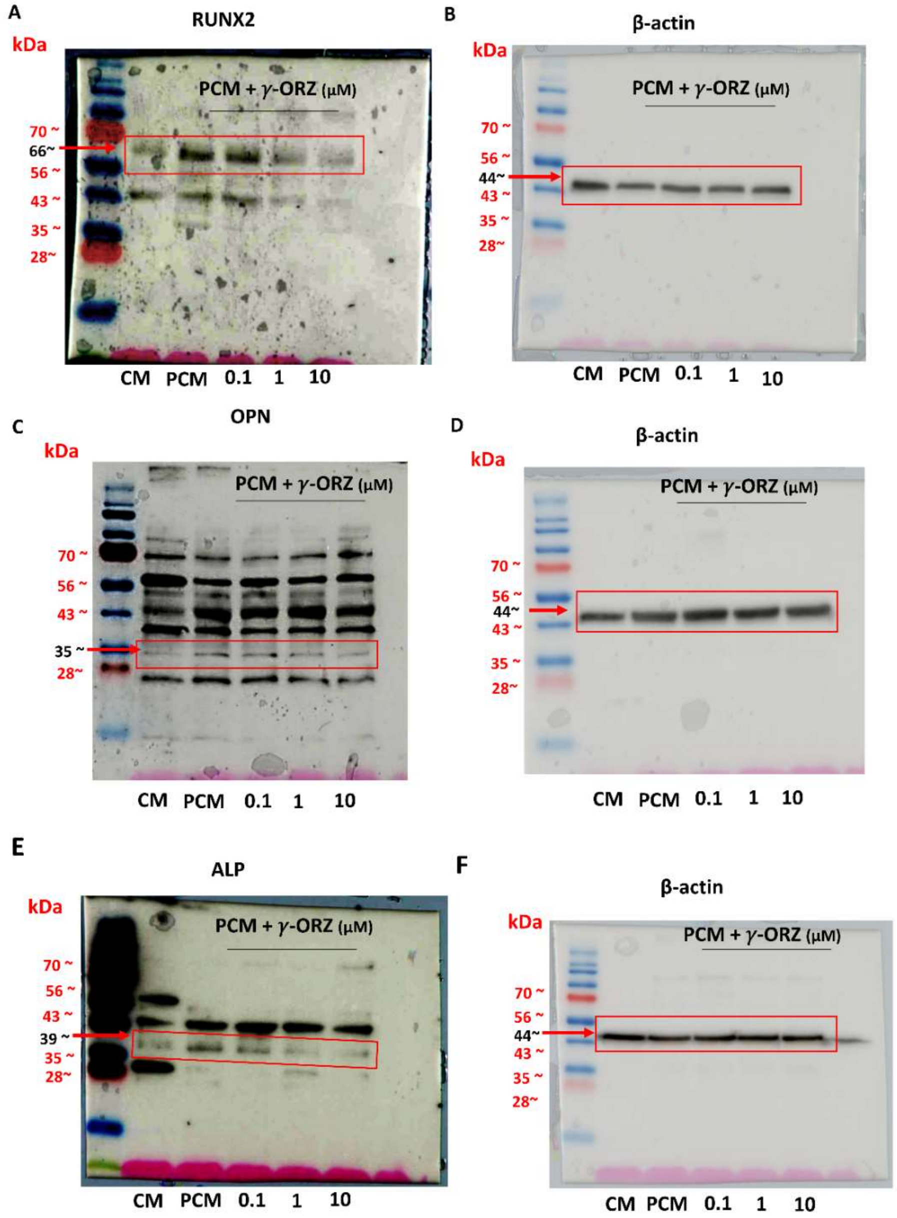

Figure S1: ***Representative full-length uncropped Western blot images corresponding to Figure 2C (RUNX2), 2D (OPN) and 3D(ALP).***

25 μg of protein loaded per lane and separated on 12% SDS-PAGE gels. (A) RUNX2, (B) β-actin for RUNX2, (C) Osteopontin (OPN), (D) β-actin for OPN, (E) Alkaline phosphatase (ALP), and (F) β-actin for ALP. β-actin was detected separately for each membrane after stripping. Data are representative of three independent experiments (n = 3). Molecular weights (kDa) are indicated on the left.

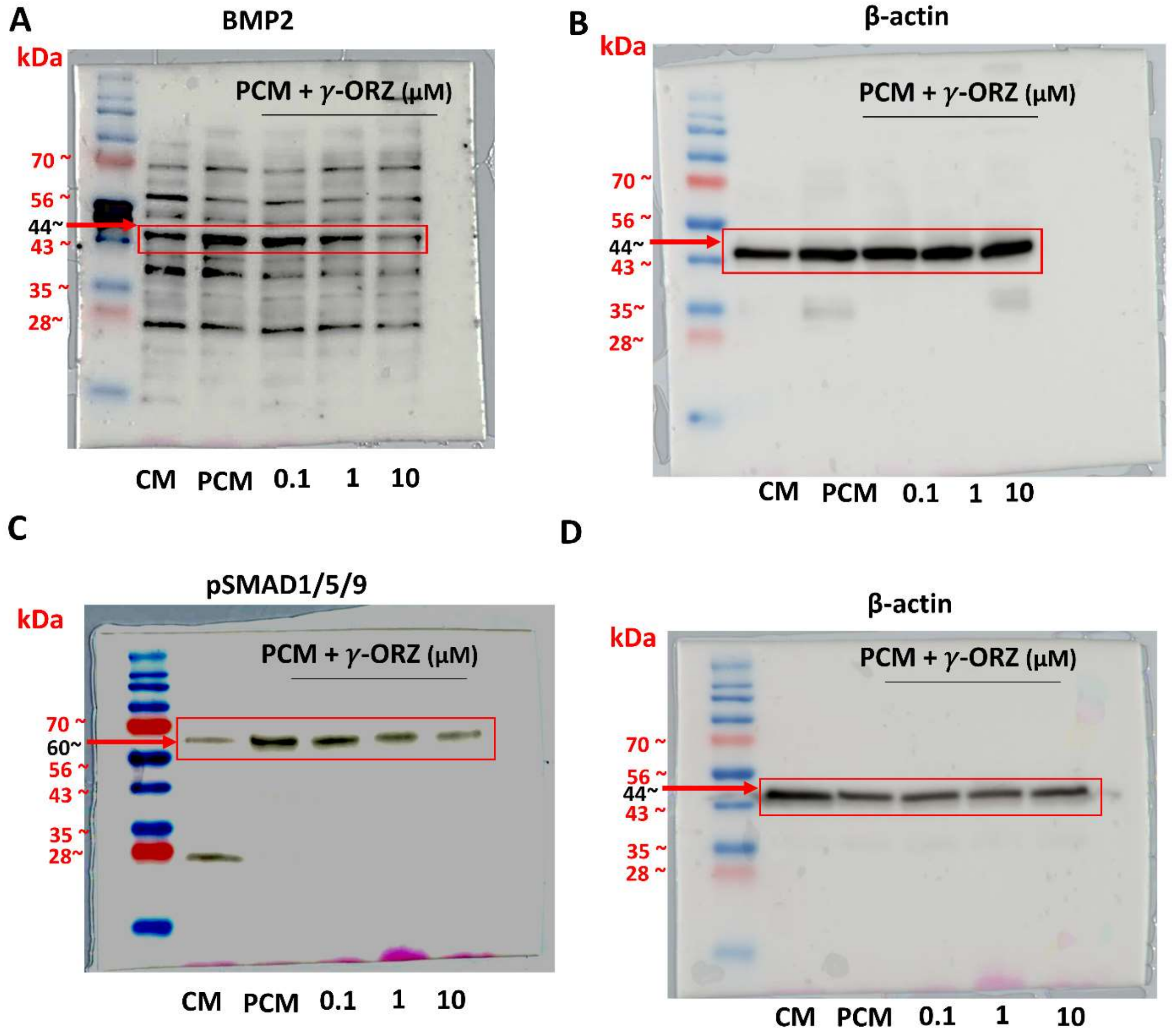

Figure S2: ***Representative full-length uncropped Western blot images corresponding to Figure 4C (BMP2), 4D (pSMAD1/5/9)***

25 µg of protein loaded per lane and separated on 12% SDS-PAGE gels. (A) BMP2 and (B) β-actin for BMP2. (C) Phosphorylated SMAD1/5/9 (pSMAD1/5/9) and (D) β-actin for pSMAD1/5/9. β-actin was detected separately for each membrane after stripping. Data are representative of three independent experiments (n = 3). Molecular weights (kDa) are indicated on the left.

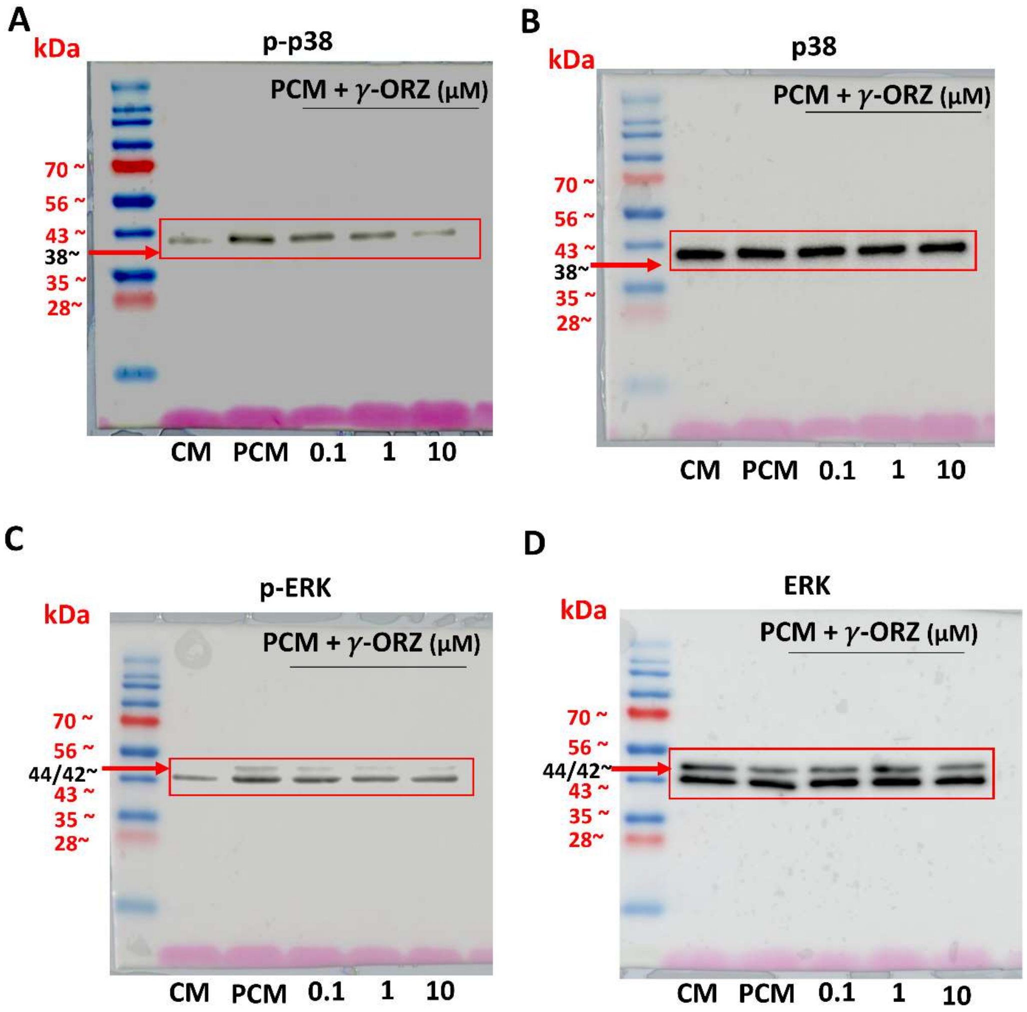

Figure S3: ***Representative full-length uncropped Western blot images corresponding to Figure 5A (p-p38 and p38) and 5B (p-ERK and ERK).***

25 μg of protein was loaded per lane and separated on 12% SDS-PAGE gels. (A) Phosphorylated p38 (p-p38) and (B) total p38; (C) phosphorylated ERK (p-ERK) and (D) total ERK. Total proteins were detected on the same membrane after stripping. Data are representative of three independent experiments (n = 3). Molecular weights (kDa) are indicated on the left.
